# Impacts and Risk Assessments of Climate Change for the Yields of the Major Grain Crops in China, Japan, and Korea

**DOI:** 10.3390/foods13060966

**Published:** 2024-03-21

**Authors:** Jieming Chou, Haofeng Jin, Yuan Xu, Weixing Zhao, Yuanmeng Li, Yidan Hao

**Affiliations:** 1 Key Laboratory of Environmental Change and Natural Disaster, MOE, Beijing Normal University, Beijing 100875, China; 202221051189@mail.bnu.edu.cn (H.J.); xuyuan01@mail.bnu.edu.cn (Y.X.); 201921051146@mail.bnu.edu.cn (W.Z.); 202231051098@mail.bnu.edu.cn (Y.L.); 202121051159@mail.bnu.edu.cn (Y.H.); 2 Institute of Disaster Risk Science, Faculty of Geographical Science, Beijing Normal University, Beijing 100875, China; 3 Southern Marine Science and Engineering Guangdong Laboratory (Zhuhai), Zhuhai 519000, China

**Keywords:** China, Japan and Korea, climate change, crop yields, risk

## Abstract

Climate change poses a high risk to grain yields. Maize, rice, and wheat are the three major grain crops in China, Japan, and Korea. Assessing the impacts and risks of climate on the yields of these grain cops is crucial. An economy–climate model (C-D-C model) was established to assess the impacts of climate factors on the grain yields in different crop areas. The peaks over threshold model based on the generalized Pareto distribution was used to calculate the value at risk and the expected shortfall, which can evaluate the yield risk of different crops. The impact ratio of climate change was employed to estimate the impacts of climate change under different climate scenarios. The main conclusions can be summarized as follows: the impacts of climate factors on grain yields and the risk vary widely across the different regions and crops. Compared to 1991–2020, climate change from 2021 to 2050 exerts positive impacts on rice and wheat, while the negative impacts on maize in the crop areas are significantly affected by climate factors. The impact ratios of climate change are larger in the SSP1-2.6 and the SSP5-8.5 scenarios than under the SSP2-4.5 scenario. These findings are useful for targeting grain yields in smaller study areas.

## 1. Introduction

The increased surface temperatures and the increased frequency and intensity of extreme events due to climate change have impacted grain crop growth, with predominantly negative effects [1]. The *Sixth Assessment Report* (AR6) of the Intergovernmental Panel on Climate Change (IPCC) promoted eight representative key risks (RKRs), including food security [1]. Grain security is an important component of food security and is related to the basic lives and livelihoods of people. Grain production is the basis for grain security. Climate change affects the yields of various types of crops positively or negatively in different regions in multiple ways [1,2]. From the perspective of the results of agricultural production activities, the risk of grain production means the uncertainty in the per unit yield or total production reduction [1,2]. From the viewpoint of the process mechanism, the risk of grain production originates from the dynamic interaction among climate-related hazards (i.e., risk-causing factors), the exposure of grain crops to hazards and the vulnerability of grain crops to hazards [1]. Within the context of climate change, the risks to grain production are becoming increasingly prominent. Volatility in grain production can lead to an insufficient food supply, trigger grain price volatility, affect trade flows, and impact the livelihoods of people, resulting in systemic risks [3].

The assessment of grain production risks is important for adjusting agricultural activities, developing preventive measures, and mitigating loss damage. Hazards have been categorized as emergent and gradual hazards [4]. Currently, risk assessment of emergent hazards receives widespread attention. Emergent hazards are often extreme weather and climate events. The event process is of short duration and can produce loss or damage in a short period of time, e.g., floods [5,6] and droughts [7]. There are two main types of risk assessment methodologies for emergent hazard generation. One is to consider risk as a function of the danger of climate hazards, as well as the vulnerability and exposure of the crops [4,8,9]; the other one is to model yields or losses using a probability distribution [6,10,11,12,13]. The assessment methods based on hazards, vulnerability, and exposure are based on the connotation of risk in the construction of indicator systems and assessment models [14,15]. Indicators are first selected or constructed to assess the above three factors, after which a yield risk function for these three factors is established [6,16,17,18]. This type of method can capture the mechanisms of grain production losses but tends to focus only on single consistent risk factors. Few studies have focused on systemic compound risks. This may be due to the complexity of the formation mechanisms of systemic compound risks. However, the methods based on the probability distribution of yields focus on directly expressing the risk magnitude from the perspective of the final impact, without considering the loss mechanism, overlooking the complex mechanisms of systemic compound risks. This class of methods can be further divided into three main categories [19]. The first category aims to fit a probability density distribution function of the yield per unit area and to calculate the probability of occurrence in both good and bad years. The second category aims to select indicators to represent the level of interannual fluctuations in the yield per unit area. Holst et al. considered the variance in the yield per unit area as a risk factor and established a flexible nonlinear fixed-effects panel data model that can be used to separately analyze the marginal contributions of climate factors to the average yield and yield risk [10]. They found that climate change affects grain production differently in northern and southern China [10]. Tigchelaar and Finger et al. analyzed the effect of climate change on crop yield variability by calculating the coefficient of variation (CV). Tigchelaar et al. examined warming-induced changes in the variability of the maize yield per unit area and concluded that future warming increases the likelihood of globally synchronized maize production shocks [13]. Finger et al. explored the impacts of climate change scenarios on the variability of maize and winter wheat yields on the Swiss Plateau [20]. The third category involves the exceedance probability of yield reduction. The exceedance probability refers to the probability that the yield reduction (loss) exceeds a certain threshold. Stojanovski et al. simulated the weather crop index (WCI) and used burn yield analysis to calculate the distribution of the rice yield loss in Hunan Province, and they obtained an aggregate exceeding probability function (AEP) curve [12]. Based on grain yield data, the method of probability statistics is used in the above research to evaluate the risk to the grain yield. However, the distribution of grain losses often exhibits non-normal and thick-tail characteristics, and the tail contains much information. There is little research on modelling the tail data of production reduction. In addition, most large-scale impact and risk assessment studies do not distinguish between various grain crop types. In fact, different grain crop yields exhibit different dimensions and should not be simply summed.

China, Japan, and Korea impose an enormous influence in northeast Asia, and grain production exerts a notable influence on society and the economy in northeast Asia. The aim of this paper is to evaluate the impacts of climate factors and future climate change on the grain yields of these three countries and the risk of grain production reduction in the current climate state. Rice, wheat, and maize were selected in this paper. In impact evaluation, the model constructed considered both socioeconomic factors and climate factors. In risk assessment, this paper focused on the data tail characteristics to ensure more accurate assessment results. The conclusions could provide a reference for the risk management of grain production in these three countries.

## 2. Materials and Methods

### 2.1. Overview of the Study Area

**Figure 1:**
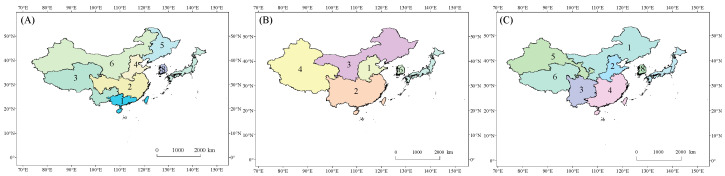
Scope and division for rice (**A**), wheat (**B**), and maize (**C**) of the study area. The numbers on the map indicate the crop areas listed in Table 1.

China, Japan, and Korea are the three most influential countries in northeast Asia (Figure 1). In 2021, the GDPs of China, Japan, and Korea were USD 17,734,062.65, USD 4,940,877.78, and USD 1,810,955.87, respectively, which were at the forefront of northeast Asia (data source: The World Bank, https://data.worldbank.org/indicator/NY.GDP.MKTP.CD, accessed on 5 July 2023). The populations of China, Japan, and Korea were 1412.36 million, 125.68 million, and 51.74 million people, respectively. The above three countries are greatly affected by the East Asian monsoon. Instability of the East Asian monsoon results in an unstable climate environment and large climate variability in these three countries.

### 2.2. Division of Crop Areas and Determination of the Growing Period

We do not further divide crop areas in Japan and Korea because the national territorial area and internal differences in the natural environment of these two countries are relatively small. We further divide the crop areas in China because the internal differences in the natural environment are large. Since it is difficult to obtain statistical data at the municipal and county levels, referring to existing research [21,22,23,24,25,26], the crop calendar of the U.S. Department of Agriculture (USDA, https://ipad.fas.usda.gov/ogamaps/cropcalendar.aspx, accessed on 15 March 2023), and the crop calendar of the Food and Agriculture Organization of the United Nations (FAO, https://cropcalendar.apps.fao.org/, accessed on 15 March 2023), we divide the 34 provincial-level administrative units of China into different crop areas. The crop areas are listed in Table 1.

The water and heat conditions in the different crop areas are different. The growing periods of crops also differ. The growing period in each crop area is determined by referring to existing research [27] and the crop calendar of the USDA (https://ipad.fas.usda.gov/ogamaps/cropcalendar.aspx, accessed on 15 March 2023) (Table 1).

### 2.3. Data and Their Sources and Preprocessing Steps

The research data consist of three main categories: statistical data, historical meteorological data, and future meteorological data.

The statistical data include the year-by-year crop production, number of people employed in agriculture, area sown, and amount of fertilizer applied by crop area from 1991 to 2020. The data were obtained from the *China Rural Statistical Yearbook, 60 Years of Statistics on Agriculture in New China* (https://zdscxx.moa.gov.cn:9443/misportal/public/publicationRedStyle.jsp (accessed on 1 March 2023)), statistical yearbooks of China’s provincial administrations, and the Food and Agriculture Organization of the United Nations (FAO, https://www.fao.org/faostat/en/#data (accessed on 1 March 2023)). The Chinese statistical data are based on provincial-level administrative districts. There are four variables collated: grain yields (i.e., grain production per unit area, kg/ha), fertilizer application per unit area (kg/ha), sown area (thousands of hectares), and population employed in agriculture (10,000 persons). Missing statistical data were interpolated. Regarding missing records in the middle of the series, the k nearest-neighbor (KNN) method was used for interpolation, with k = 1. Regarding missing grain data, the gaps were filled according to the relationship between the yield, sown areas, and total yield. In regard to missing records at the end of the series of the population employed in agriculture in each province of China, different treatments were adopted according to the different conditions. The Augmented Dickey–Fuller (ADF) unit root test was used to determine whether the data series of the population employed in agriculture in all provinces exhibits smoothness after first-order differencing. The LB statistic was then used to determine whether the differenced time series was a white noise series. If the series was a nonwhite noise series, the ARIMA model was employed to supplement any missing data (due to the reform of administrative divisions, missing data in the tail of the data of Sichuan were estimated by subtracting the tail data of Chongqing from the sum of the data of Sichuan and Chongqing.) If the series was a white noise series, the curve-fitting method was used to fit the original series for estimation purposes. The equations of the fitted curves are provided in Table A1.

The historical meteorological data include the average air temperature (°C) and downward shortwave radiation flux density received at the surface (W/m^2^) during the growing period in each crop area from 1991 to 2020. Temperature data were obtained from the CRU TS dataset version 4.06 (https://crudata.uea.ac.uk/cru/data/hrg/cru_ts_4.06/cruts.2205201912.v4.06/ (accessed on 1 March 2023)). These data were obtained via interpolation of the station data with an accuracy of 0.5° × 0.5°. The downward shortwave radiation flux density received at the surface was obtained from the variable “surface solar radiation downwards (in J/m^2^)” in the ERA5-Land monthly averaged data from 1950 to present (https://cds.climate.copernicus.eu/cdsapp#!/dataset/10.24381/cds.e2161bac?tab=overview (accessed on 1 March 2023)). The accuracy of the data is 0.1° × 0.1°, and the data are reanalyzed data. The average of all gridded points within a given crop area was used as the value for this crop area. The values of the above climate factors for each month during the growing season were first calculated, and the average was then obtained.

The future meteorological data include the mean air temperature (°C) and downward shortwave radiation flux density received at the surface (W/m^2^) during the growing period in each crop area from 2021 to 2050 under three scenarios (SSP1-2.6, SSP2-4.5, and SSP5-8.5). SSP1-2.6 represents a world with sustainable development and a low climate change challenge. Under this scenario, the global CO_2_ emissions significantly decrease, reaching net zero levels after 2050. SSP2-4.5 represents the intermediate pathway, with a medium climate change challenge. CO_2_ emissions fluctuate at current levels before beginning to decline at the middle of the century but do not reach net zero levels before 2100. SSP5-8.5 emphasizes the traditional economic orientation, with a significant increase in CO_2_ emissions. The NASA Earth Exchange Global Daily Downscaled Projections (NEX-GDDP-CMIP6) dataset (https://doi.org/10.7917/OFSG3345 (accessed on 1 August 2023)) provides a set of global, high-resolution, bias-corrected climate change projections that can be used to assess the impact of climate change on processes sensitive to small-scale climate gradients and the influence of local topography on climatic conditions [28,29,30]. In this paper, we calculated the equally weighted average of all models listed in Table A2 as the projected data of the different future scenarios with a resolution of 0.5° × 0.5°.

### 2.4. Methods

First, principal component scores were calculated using principal component analysis to obtain the comprehensive climate factor (CCF) for each crop area [31]. The output elasticity of the climate change factor was estimated by regarding the CCF as a climate change factor input of the climate–economic model (C-D-C model) [32,33,34]. Based on the climate yield loss data, the peaks over threshold (POT) model based on the generalized Pareto distribution (GPD) was used to calculate the value at risk (VaR) and the expected shortfall (ES) to assess the risk of the yields of the different crops in the current climatic state. We adopted three scenarios, i.e., SSP1-2.6, SSP2-4.5, and SSP5-8.5, and calculated the CCF from 2021 to 2050 under each scenario. The impacts on grain yields under the different climate scenarios were estimated using the impact ratio of climate change (IRCC) [35,36,37].

#### 2.4.1. Comprehensive Climate Factor

We obtained the CCF by extracting the main information of multiple climate factors through principal component analysis (PCA) [31]. This index can be regarded as an input factor of climate production in the C-D-C model. The specific calculation steps are as follows:

Step 1. Choose the climate factors and the standardization approach.

When selecting climate factors, careful consideration should be given to the selection of precipitation. Irrigation is a technical measure that has been used since ancient times to resolve the lack of natural precipitation during agricultural activities. The amount of precipitation often does not reflect the actual amount of water received by crops in crop areas. For example, since the 1990s, China has achieved remarkable progress in water-saving technology, gradually improved policy and institutional safeguards, and significantly increased their investment in irrigation water conservancy projects [38]. Three climate factors, namely, the mean air temperature, cumulative precipitation, and downward shortwave radiation flux density received at the surface, were first selected to calculate the CCF; then, only the mean air temperature and downward shortwave radiation flux density received at the surface were selected to calculate the CCF. The CCF values obtained in these two ways were substituted into the C-D-C model, and the effects of the two CCF calculation methods were compared according to the adjusted R^2^ and mean relative error values. Finally, two climate factors, namely, the average air temperature (°C) and the downward shortwave radiation flux density received at the surface (W/m^2^), were selected to calculate the CCF.

When calculating the value of a climate factor for a given year, we should select appropriate months instead of all months of the year [32,33]. In this paper, the average values of the climate factors during the growing period were calculated.

The method of calculating the CCF is described below using an example from one crop area of a certain crop.

Step 2. Use a PCA on standardized variables with an 80% threshold to obtain the principal component score *z_ki_*, which is the score of the *i*th principal component in the *k*th year (1991–2020).

Step 3. Calculate the *CCF*.

(1) $$CCF_{k}=\sum_{i=1}^{n}\left(z_{ki}\times \frac{\alpha_{i}}{\sum_{i=1}^{n}\alpha_{i}}\right)+20$$

where *α_i_* is the variance contribution rate of the *i*^th^ principal component.

The production factors in the C-D-C model must be positive [32]. To ensure that the *CCF* is positive, it is shifted upwards by 20 units overall.

#### 2.4.2. C-D-C Model

The C-D-C model is based on the C-D production function model proposed by Cherles Cobb and Paul Dauglas to introduce climate production input factors [33]. The C-D-C production function represents the relationship between production and inputs for a given technology. The C-D-C model was used to estimate the effect of each production factor on grain yields [32,33].

(2) $$y=x_{1}^{\beta_{1}}x_{2}^{\beta_{2}}x_{3}^{\beta_{3}}C^{\gamma }\mu$$

The terms at the right of Equation (2) represent the production inputs. *x_i_* (*i* = 1, 2, 3) is a socioeconomic input; *C* is a climate input; and *μ* represents the other unconsidered factors. The amount of the socioeconomic input factors is not necessarily equal to 3, but each input needs to have a certain amount in regional farming. The terms at the left of Equation (2) represent the economic output. Other parameters are used to show the functional relationship between the input and the output. In order to estimate these parameters, calculate the logarithm of both sides of Equation (2); the nonlinear function can be transformed into a linear function, yielding Equation (3):

ln(*y*) = *β*_1_ln(*x*_1_) + *β*_2_ln(*x*_2_) + *β*_3_ln(*x*_3_) + *γ*ln*C* + *μ*′ (3)

where *y* is the grain yield (kg/ha); *x*_1_, *x*_2_, and *x*_3_ are the population employed in agriculture (10,000), the sown area (10^3^ ha), and the fertilizer application amount per unit area (kg/ha), respectively; *C* is the climate production input factor—the *CCF* is used here; *β*_1_, *β*_2_, *β*_3_, *γ*, and *μ*′ are the coefficients to be estimated; and *β*_1_, *β*_2_, *β*_3_, and *γ* denote the output elasticity of the factors, indicating the corresponding percent increase in the yields for a 1% increase in the production factor.

The multiple linear regression model assumes that the dependent variable follows a normal distribution. Therefore, the Kolmogorov–Smirnov test was used to perform the normality test of ln(*y*). The partial regression coefficients of Equation (3) were estimated using the ordinary least squares (OLS) method. The F-test was applied to the model to determine the significance of the linear relationship between all independent variables and the dependent variable. A *t*-test was performed using the partial regression coefficients to determine whether the linear relationship between each independent variable and the dependent variable was significant. The residual sum of squares (RSS) and adjusted R^2^ values were calculated to assess the fit of the model. A total of 6 leave-5-out cross-validation steps were performed, and the mean of the relative error was calculated to evaluate the extrapolated predictive ability of the model.

#### 2.4.3. Impact Ratio of Climate Change

The impact ratio of climate change (IRCC) can be used to project the influence of future climate change on grain yields.

In Equation (2), let $N=x_{1}^{\beta_{1}}x_{2}^{\beta_{2}}x_{3}^{\beta_{3}}\mu$, then $y=N_{C}C^{\gamma }$.

It is assumed that the average conditions from 1991 to 2020 are *y*_1_, *N*_C1_, and *C*_1_, and the average conditions from 2021 to 2050 are *y*_2_, *N*_C2_, and *C*_2_, respectively. Let the following apply:

(4) $$y_{1}=N_{C1}C_{1}^{\gamma },$$

(5) $$y_{2}=N_{C2}C_{2}^{\gamma },$$

(6) $$y^{*}=N_{C2}C_{1}^{\gamma }$$

where *y*^∗^ is the yield under a hypothetical scenario, which is the grain yield under the socioeconomic level (*N*_C_) from 2021 to 2050 and the climate state (*C*) from 1991 to 2020. Then, by subtracting Equation (6) from Equation (5), the part of grain yield only affected by climate change was obtained, which is called the yield impact of climatic change (YICC) Δ*y*.

(7) $$\Delta y=y_{2}-y^{*}=\frac{C_{2}^{\gamma }(N_{C2}C_{2}^{\gamma }-N_{C2}C_{1}^{\gamma })}{C_{2}^{\gamma }}=y_{2}\cdot \frac{C_{2}^{\gamma }-C_{1}^{\gamma }}{C_{2}^{\gamma }}$$

An algebraic transformation of Equation (7) was carried out to obtain Equation (8), which can be defined as the impact ratio of climate change.

(8) $$IRCC=\frac{\Delta y}{y_{2}}=\frac{C_{2}^{\gamma }-C_{1}^{\gamma }}{C_{2}^{\gamma }}$$

The *IRCC* denotes the proportion of the direct impact of climate change on grain yields in the actual yields. It is a benefit index to measure the impact of climate change on the economic output. Using this index, the sensitivity of output changes to climate change in three scenarios (SSP1-2.6, SSP2-4.5, and SSP5-8.5) can be analyzed.

#### 2.4.4. Climate Yields and Climate Loss

Actual yields consist of trend yields, climate yields, and random yields [39]:

*y*_actual_ = *y*_trend_ + *y*_climate_ + *y*_random_ (9)

where *y*_actual_, *y*_trend_, *y*_climate_, and *y*_random_ denote the actual yields, trend yields, climate yields, and random yields, respectively.

Trend yields, also known as technical yields, respond to yields due to the level of technology. Trend yields were calculated using four methods for the yield time series: sliding 5-year average, sliding 3-year average, linear regression, and exponential regression. These 4 climate yields are basically the same in each trend. Therefore, the average of these trend yields calculated using the above four methods was chosen as the final trend yield. The grain yield of a region in a given year is mainly determined using two factors: technology and climate. The random yield can be ignored. Therefore, climate yields can be calculated via Equation (10), which indicates the contribution of climate factors to the yield.

*y*_climate_ = *y*_actual_ − *y*_trend_ (10)

Climate loss (*y*_loss_) is the opposite of climate yields. It is the yield amount lost due to climate factors.

#### 2.4.5. POT Model Based on the GPD

Climate loss data usually exhibit a thick tail that contains a wealth of information. Therefore, tail modelling of climate loss is necessary. The peaks over threshold (POT) model based on the generalized Pareto distribution (GPD) was used to calculate the value at risk (VaR) and expected shortfall (ES) for estimating the risk of climate yield loss of the major grain crops in China, Japan, and Korea in the climate state from 1991 to 2020.

Step1. Assess the thick tail.

Modelling data tails requires the data to exhibit thick tails. Thick-tailedness was assessed by plotting a quantile–quantile plot (Q–-Q plot) of the standard exponential distribution [40,41]. If the scatter points were convex upwards overall, the data could be considered to exhibit thick tails. Upon testing, we found that the data of all crop areas exhibited thick tails except the climate loss data of the winter wheat (autumn sowing) area in southern China. Therefore, the POT model based on the GPD could not be used to calculate the VaR and ES for the winter wheat (autumn sowing) area in southern China. A normality test of the data of this crop area was conducted. It passed the Kolmogorov–Smirnov test with *p* ≈ 0.9126. We accepted the original hypothesis that the data obeyed a normal distribution.

Step 2. Fit the probability distribution and estimate the parameters.

Set a threshold *μ*. The POT model can only be modelled for the excess value of the data beyond the threshold. It is assumed that *N_μ_* samples of the data are greater than the threshold (*N_μ_* < *n*), where *n* is the number of sample data (*n* = 30). The excess value of the data can be calculated as follows:

*Y_i_* = *y*_loss,*k*_ − *μ*, *i* ∈ {1, 2, …, *N_μ_*}. (11)

The conditional excess distribution function *F_μ_*(*Y*) above this threshold is

(12) $$F_{\mu }(Y)=\frac{F(\mu +Y)-F(\mu )}{1-F(\mu )}.$$

When *µ* is sufficiently large, it can be well approximated by the GPD regardless of the form of the base distribution [40,41]. The parameters *ξ* and *β* were estimated using the maximum likelihood estimation (MLE) method [42]:

(13) $$G(Y)=\left\{ \begin{array}{l} 1-{\left(1+\frac{Y\xi }{\beta }\right)}^{-\frac{1}{\xi }},\xi \neq 0\\ 1-e^{-\frac{Y}{\beta }},\xi =0 \end{array}\right.$$

where *ξ* is a shape parameter, *β* is a scale parameter, and *β* > 0.

Ther thresholds were determined by combining the mean exceedance function method and the kurtosis coefficient method [43,44]. The mean exceedance function method determines the threshold based on the mean residual life plot of the sample data. The mean exceedance function *e*(*μ*) can be expressed as

(14) $$e(\mu )=E(y_{loss}-\mu |y_{loss}>\mu )=\frac{\sum_{i=1}^{N_{\mu }}\left(y_{loss,i}-\mu \right)}{N_{\mu }}.$$

The mean residual life plot was generated with *μ* as the horizontal axis and *e*(*μ*) as the vertical axis. The appropriate *μ*_0_ value was selected as the threshold so that *e*(*μ*) is approximately linear for *μ* ≥ *μ*_0_. The kurtosis coefficient method is based on using the kurtosis *K* to determine the coefficient. First, the kurtosis of the sample data was calculated. For *K* ≥ 3, the maximum *y*_loss,*k*_ of the data was removed from the samples. This process was repeated until *K* < 3 was obtained. The maximum *y*_loss,*k*_ from the remaining samples was chosen as the threshold. Having combined the above two methods to determine the threshold, the results are shown in Table 2.

Step 3. Calculate the VaR and ES values.

VaR is the maximum possible climate loss in a specific period of time in the near future at a certain confidence level. Compared with VaR, ES adds more information. ES is defined as the conditional expectation value of VaR for which the loss data is greater than confidence *p*. According to the previous derivation, *VaR* and *ES* were calculated according to Equations (15) and (16) [45], respectively.

(15) $$P(y_{loss}>VaR)=1-p,VaR=\mu +\frac{\beta }{\xi }\{{\left[\frac{n}{N_{\mu }}(1-p)\right]}^{-\xi }-1\}$$

where *p* = 0.05.

(16) $$\begin{array}{l} ES=VaR+E(y_{loss}-VaR|y_{loss}>VaR)=E(y_{loss}|y_{loss}>VaR)\\ =VaR+\frac{\beta +\xi (VaR-\mu )}{1-\xi } \end{array}$$

## 3. Results

### 3.1. Impacts of the Climate Factors on Grain Yields

#### 3.1.1. OLS Estimation Results

Output elasticities were estimated for each production factor using the C-D-C model for each cropping area. The logarithms of the yield series for all crop areas passed the Kolmogorov–Smirnov normality test, which indicates that the coefficients could be estimated using the OLS method. All models for the rice crop areas demonstrated that the original hypothesis of the F-test could be rejected at the 0.05 significance level, which indicated that the model was significant. All models for the wheat crop areas except for the Korean wheat crop areas suggested that the original hypothesis of the F-test could be rejected at the 0.01 significance level, which suggested significance of the models. All models for the maize crop areas demonstrated that the original hypothesis of the F-test could be rejected at the 0.01 significance level, which denoted a significant model. The residual sum of squares and adjusted R^2^ were calculated, and the leave-5-out test was performed. The results are listed in Table 3, revealing that the models were fitted and extrapolated well.

**Figure 2:**
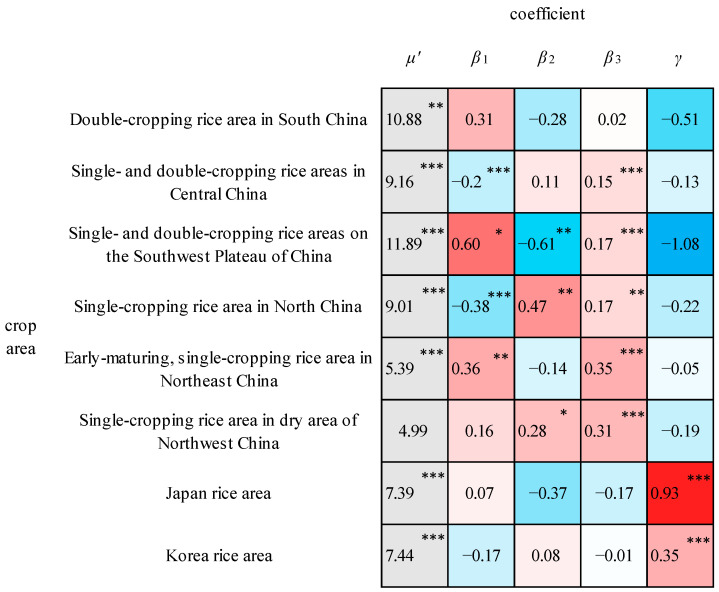
Partial regression coefficients for the rice crop areas. ***, **, and * indicate that the coefficient is significant at 1%, 5%, and 10%, respectively.

The output elasticities of the production factors in each rice crop area are shown in Figure 2. The climate output elasticities (*γ*) for Japan and Korea passed the significance test, which demonstrated that the climate factors impose a significant effect on the yields in these two crop areas. The climate output elasticity for Japan’s rice area is 1.88, which indicates that a 1% increase in the CCF increases rice yields by 1.88%. The climate output elasticity for Korea is 0.61, indicating that a 1% increase in the CCF causes an increase in rice yields of 0.61%. Only *γ* is significant in the partial regression coefficients for the Japan rice area and Korea rice area with large absolute values, while the partial regression coefficients of the other economic and social factors are not significant. This demonstrates that the rice yields in Japan and Korea are mainly influenced by climatic factors such as temperature and solar radiation. Rice is a crop that is sensitive to climatic conditions. For example, increased temperatures can lead to heat stress at critical growth stages, affecting pollen vigor and leading to yield reductions. Japan and Korea are coastal countries with abundant water vapor, high air humidity, and high cloudiness, which can reduce the amount of solar radiation received at the surface and limit the photosynthesis of rice. Rising temperatures combined with higher humidity may increase crop pests and diseases [46]. Japan and Korea are located in monsoon climate zones with unstable climatic environments that are prone to extreme weather events such as typhoons and persistent heavy precipitation. These types of extreme weather events can cause flooding and waterlogging, which, in turn, can lead to yield reductions [47]. In addition, typhoons and persistent precipitation can reduce the solar radiation flux density received at the surface.

In contrast, the output elasticities of the climatic factors were not significant in the rice crop areas of China, while the output elasticities of the socioeconomic factors were significant in most crop areas. This suggests that the rice yields in most Chinese crop areas are influenced by socioeconomic factors, with less influence resulting from climate. The output elasticities of the fertilizer application amount per unit area (*β*_3_) passed the significance test at the 0.05 significance level, with positive values for all crop areas in China, except for the double-cropping rice area in South China. This demonstrates that an appropriate increase in the amount of fertilizer applied can help to improve the rice yields in China. The increasing technology level reduces the sensitivity of rice production to climate and improves its adaptability to different climate environments. The coefficients of the sown area (*β*_2_) of the three crop areas—single- and double-cropping rice areas on the Southwest Plateau of China, single-cropping rice area in North China, and single-cropping rice areas in dry area of Northwest China—are significant. These three crop areas exhibited small sown areas relative to the other crop areas from 1991 to 2020. In contrast, the sown area of the crop areas with a statistically insignificant elasticity coefficient (*β*_2_) was larger than the sown area of the other crop areas. The elasticity coefficients of the population employed in agriculture (*β*_1_) were significantly negative in the single- and double-cropping rice areas in Central China and the single-cropping rice areas in North China, which suggests that there exists a relationship between the decrease in agricultural labor and the increase in rice yields. This may occur because the improvement in planting technology in these two crop areas released the agricultural labor force and increased the level of yields at the same time. Moreover, the fact that the agricultural labor inputs in these two crop areas were higher than those in the other crop areas from 1991 to 2020 and generally showed a decreasing trend may support this explanation. In addition, although the level of agricultural mechanization and technology was higher in the early-maturing, single-cropping rice area in Northeast China, it still faced labor shortages, and *β*_1_ was significantly positive.

**Figure 3:**
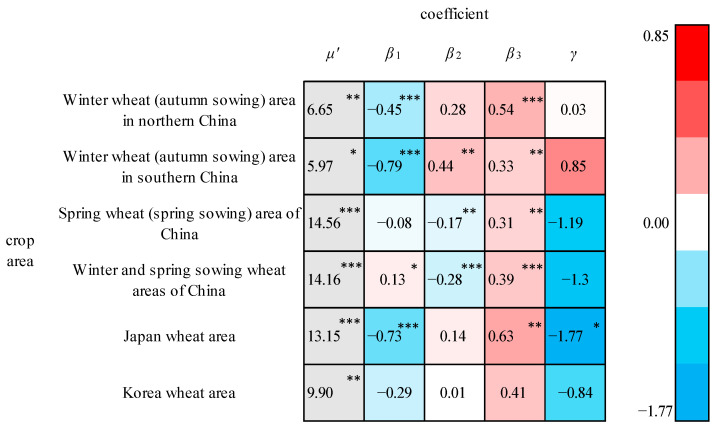
Partial regression coefficients for the wheat crop areas. ***, **, and * indicate that the coefficient is significant at 1%, 5%, and 10%, respectively.

The output elasticities of the wheat production factors for each crop area are shown in Figure 3. Only the climate output elasticity (*γ*) of the Japan wheat region passed the significance test at a significance level of 0.1, which indicates that the wheat yields are mainly influenced by socioeconomic factors instead of climate factors. This probably occurs because wheat is hardy and eurythermal. The elasticity coefficients of the fertilizer application amount per unit area (*β*_3_) were significant at the 5% significance level for all wheat cropping regions except Korea, and their values were greater than 0.3, which is relatively large. This demonstrates that the wheat yields are significantly affected by the amount of fertilizer applied. The elasticities of the number of people employed in agriculture (*β*_1_) for the winter wheat (autumn sowing) area in northern China, the winter wheat (autumn sowing) area in southern China, and the Japan wheat area were negative. This may be due to the existence of a factor that is negatively correlated with agricultural labor and positively correlated with the yields in these three regions, such as the level of agricultural automation and the level of agricultural mechanization, which results in a negative elasticity of labor. Owing to the improvement in the agricultural technology level, the direct impact of agricultural labor itself on the wheat yield is limited. In addition, the agricultural laborers in these three cropping regions exhibited higher inputs than those in the other cropping regions during the study period and generally showed a decreasing trend, which can also indirectly confirm this explanation.

**Figure 4:**
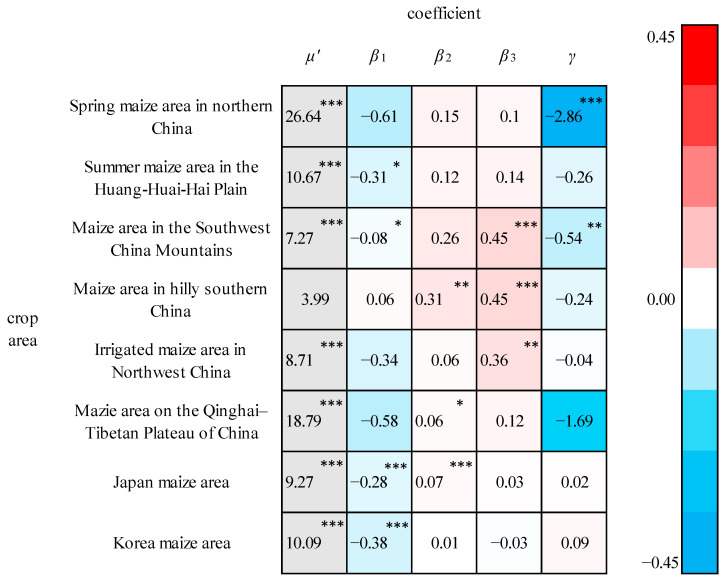
Partial regression coefficients for the maize crop areas. ***, **, and * indicate that the coefficient is significant at 1%, 5%, and 10%, respectively.

The output elasticities of the maize production factors in each crop area are shown in Figure 4. The climate output elasticities (*γ*) for the spring maize area in northern China and the maize area in the Southwest China Mountains passed the significance test, which indicated that the climate factors imposed a significant effect on the maize yields in these two crop areas. Between them, the absolute value of the elasticity of the climate factor was larger in the spring maize area in northern China, where a 1% increase in the CCF caused a decrease in maize yields of 2.86%, followed by the maize area in the Southwest China Mountains, where a 1% increase in the CCF cause a decrease in the maize yields of 0.54%. The maize yields in the spring maize areas in northern China are more sensitive than those in the maize areas in the Southwest China Mountains. Late spring frost is prone to occur in the spring maize area in northern China, which is at the end of the maize sowing period and the beginning of the growth period, affecting the growth of maize seedlings. There are few light and heat resources in the maize area in the Southwest China Mountains with high interannual variation, which exerts a notable impact on the growth of maize. The socioeconomic output elasticities for the spring maize area in northern China were not significant, which demonstrates that the maize yields in this crop area are mainly influenced by climate factors. The output elasticity of the fertilizer application amount per unit area (*β*_3_) for the maize area in the Southwest China Mountains passed the significance test at a significance level of 0.01. Thus, the loss caused by climate factors could be reduced via rational fertilization.

#### 3.1.2. Discussion of Multicollinearity

Economic data often suffer the problem of multicollinearity, which increases the variance in the biased regression coefficients and reduces the validity of the coefficients [48]. The ridge regression (RR) estimation method proposed by Hoerl and Kennard could be used to reduce the variance by sacrificing the unbiasedness of the estimation [49]. In this paper, the existence of multicollinearity was assessed by calculating the variance inflation factor (VIF) of the independent variables for each cropping area. If the VIF of some independent variable is greater than 10, the independent variable will be considered to be linearly correlated with the other independent variables, and the partial regression coefficients must be re-estimated using the ridge regression method. As indicated in Table 4, multicollinearity was found in the data of the early-maturing, single-cropping rice area in Northeast China, the Japan rice area, the Korea rice area, the spring maize area in northern China, the maize area in hilly southern China, and the irrigated maize area in Northwest China. Ridge regression analysis was used for these six crop areas, and the variance inflation factor method was used to determine the ridge parameter *k* automatically.

Compared to the coefficients estimated using the OLS, the absolute values of the climate coefficients estimated via ridge regression analysis are lower, and the coefficients of more socioeconomic factors passed the significance test (Table 5). The coefficient of the population employed in agriculture (*β*_1_) for the Korea rice area passed the significance test at the significance level of 0.05, but its absolute value is −0.07, which is very small. The coefficient of the sown area (*β*_2_) is −0.24 for the Japan rice area and only passes the significance test at the significance level of 0.10, which is weak. The ridge regression estimation results still indicate that the yields in Japan and Korea are mainly influenced by climate. The coefficient of the sown area (*β*_2_) is 0.05 for the early-maturing, single-cropping rice region in northeast China and passed the significance test at a significance level of 0.01. Among the three maize crop areas, the elasticities of the fertilizer application amount per unit area were lower in the ridge regression results than in the OLS estimates, and the number of people employed in agriculture and the area under cultivation explained more of the total variation in yields. In the three maize crop areas, the significance of the climate coefficients did not change, while the socioeconomic factors could explain more of the changes in the yields.

#### 3.1.3. Discussion of Considering Technological Advancements

Agricultural technology develops over time within a crop area. Technological advancements can be captured by adding dummy variables for a group of years into the model, reflecting shifts in the C-D-C production function due to technological improvements. The research period was divided into three stages: 1991–2000, 2001–2010, and 2011–2020. It is considered that there is no difference in technical level in each stage and there are differences between different stages.

ln(*y*) = *β*_1_ln(*x*_1_) + *β*_2_ln(*x*_2_) + *β*_3_ln(*x*_3_) + *γ* ln*C* + *b*_1_*d*_1_ + *b*_2_*d*_2_ + *μ*′ (17)

where

$$d_{1}=\left\{ \begin{array}{l} 1,\;\mathrm{the}\;\mathrm{sample}\;\mathrm{is}\;\mathrm{during}\;2001–2010.\\ 0,\;\mathrm{the}\;\mathrm{sample}\;\mathrm{is}\;\mathrm{during}\;1991–2000\;\mathrm{or}\;2011–2020. \end{array}\right.,\;d_{2}=\left\{ \begin{array}{l} 1,\;\mathrm{the}\;\mathrm{sample}\;\mathrm{is}\;\mathrm{during}\;2011–2020.\\ 0,\;\mathrm{the}\;\mathrm{sample}\;\mathrm{is}\;\mathrm{during}\;1991–2010. \end{array}\right.$$

*b*_1_ and *b*_2_ are the coefficients to be estimated.

The estimation results obtain using the OLS method are shown in Table 6. Having fixed the influence of different levels of agricultural technology, the basic results did not change. The yields in the Japan rice area, Korea rice area, Japan wheat area, Spring maize area in northern China, and maize area in the Southwest China Mountains are mainly affected by climate factors. Fertilizer application can play an important role in most Chinese crop areas.

### 3.2. Impacts of Future Climate Change on Grain Yields

**Figure 5:**
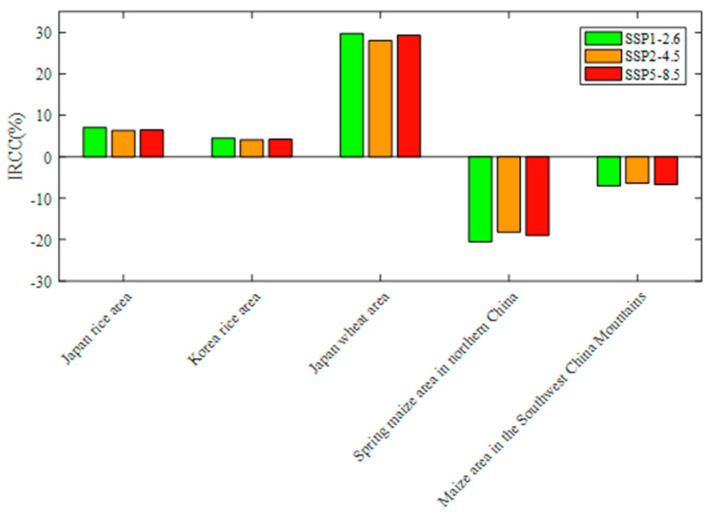
IRCC in the crop areas with significant climate impacts under the different scenarios.

The average value of the CCF from 1991 to 2020 was selected as the historical climate state, and the average value of the CCF from 2021 to 2050 was selected as the future climate state. The impact ratio of climate change (IRCC) was calculated for each crop area with a significant climate coefficient. The results are shown in Figure 5. Overall, the contribution of future climate change to future changes in grain yields is large in crop areas with significant climate impacts. The climate affects grain yields to a lesser extent under scenario SSP2-4.5 and to a greater extent under scenarios SSP1-2.6 and SSP5-8.5.

Regarding the rice crop areas and wheat crop areas that are significantly affected by climate, the impact of future climate change is mainly positive. In the maize crop areas that are significantly affected by climate change, the impact of climate change is mainly negative. Future climate change impacts on grain yields in actual yields are small in the Japan rice region, Korea rice region, and maize area in the Southwest China Mountains, whose |IRCC| is less than 10%. The weight of climate change impacts on the actual yields is high in the Japan rice area and spring maize area in northern China, whose |IRCC| is greater than 15%. This may be related to the unstable future climate environment in these two crop areas [7,50].

### 3.3. Risk Assessment of Grain Yields

#### 3.3.1. Value at Risk (VaR) and Expected Shortfall (ES)

**Figure 6:**
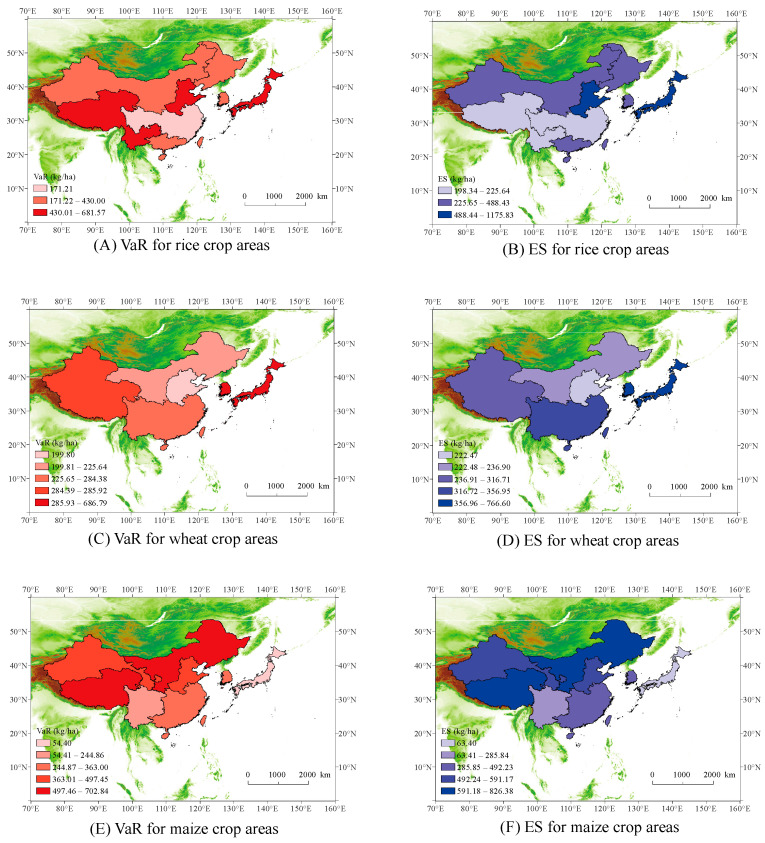
VaR for rice (**A**), wheat (**C**), and maize (**E**) crop areas. ES for rice (**B**), wheat (**D**), and maize (**F**) crop areas.

The annual climate yield loss of each crop area was calculated. A POT model based on the GPD was constructed for the tail data, and the value at risk (VaR) and expected shortfall (ES) were calculated. For rice (Figure 6A,B), the crop area with the highest risk of climate yield loss is the single-cropping rice area in north China, followed by the Japan rice area. The VaR of the single-cropping rice area in north China is 618.57 kg/ha, and the ES is 1175.83 kg/ha. The VaR of the Japan rice crop area is 562.91 kg/ha, and the ES is 934.55 kg/ha. Both north China and Japan are located in the monsoon region of East Asia, where the circulatory atmospheric circulation is unstable with high interannual variability in precipitation [39]. Typhoons are frequent in Japan, and the growing period of maize overlaps with the period of high typhoon frequency [47]. Climate warming has led to an increasing frequency and intensity of droughts in north China [39]. The crop area with the lowest risk is the single- and double-cropping rice area in central China, where the VaR and ES do not exceed 200 kg/ha. The coefficients of the population employed in agriculture and the fertilizer application amount per unit area in this crop area are significant, while the climate elasticity of the output is not significant, which demonstrates that progress in agricultural technology and an increase in the amount of fertilizer applied in this crop area may be conducive to reducing climate-induced losses. Regarding wheat (Figure 6C,D), most of the crop areas in China exhibit low risk, while those in Japan and Korea exhibit high risk. However, Japan and Korea’s grain crops are dominated by rice, so a higher risk of wheat yields would be less detrimental to local food security and their overall socioeconomic situation. Regarding maize (Figure 6E,F), the yield risk is higher in the spring maize area in northern China and the maize area on the Qinghai–Tibetan Plateau. In regard to the spring maize area in northern China, the VaR is 627.94 kg/ha, and the ES is 826.38 kg/ha. Regarding the maize area on the Qinghai–Tibetan Plateau, the VaR is 702.84 kg/ha, and the ES is 760.49 kg/ha. These findings are likely related to the unstable climate environment during the growing season in these two areas. Late spring frost often occurs in north China and northeast China. The Tibetan Plateau exhibits high altitude, low temperatures, and long growing periods. However, the accumulated temperature is usually not sufficient, resulting in unstable maturity and yields. Moreover, the large values of the climate output elasticities for these two crop areas obtained through the C-D-C model passed the significance test. This indicates that climate factors impose a greater influence and risk on the maize yields in these two crop areas.

#### 3.3.2. Sensitivity Analysis

**Figure 7:**
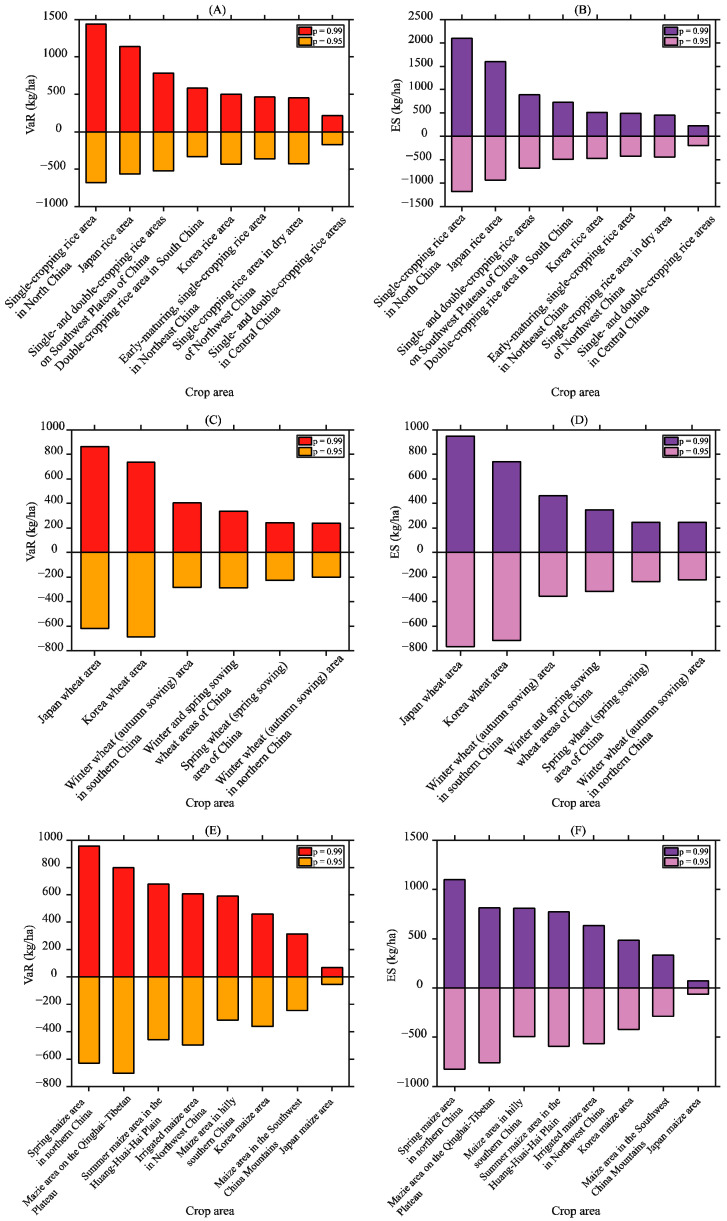
Comparison of the VaR with different *p* values in rice (**A**), wheat (**C**), and maize (**E**) crop areas. Comparison of the ES with different *p* values in rice (**B**), wheat (**D**), and maize (**F**) crop areas.

The GPD-based POT model requires setting the confidence level *p*. The VaR and ES are different at different confidence levels. The *p* value was changed from 0.95 to 0.99, and the tail data were modelled again based on the same model. The results with the new *p* value are shown in Figure 7. The VaR and ES at a confidence level of 0.99 were higher than those at a confidence level of 0.95, which indicates that the confidence level must be carefully determined. However, the rankings of the VaR and ES for each crop area of the same crop remained essentially unchanged, indicating that the model used is relatively robust.

## 4. Discussion

In this paper, three grain crops, namely, maize, rice, and wheat, were selected, and different grain crop areas were delineated based on existing studies. A climate–economic model (C-D-C model) was used to estimate the output elasticity of climate factors and to assess the impact of climate factors on the grain yields in different crop areas. The impacts of climate factors on the three major food crops in China, Japan, and Korea varied according to the different crop types and crop areas [10,38,46,51,52]. Cropping areas significantly affected by climatic factors tend to have unstable climate environments, most of which occur in monsoon climate zones. Regarding rice, the yields in the Japan rice area and the Korean rice area are mainly influenced by climate factors, while the socioeconomic impacts are not significant. In China, the rice yields are mainly influenced by socioeconomic factors and not significantly by climatic factors, which suggests that China can use socioeconomic resources to better withstand the risk of regional climate variability in rice production, including implementing measures such as investing more capital and promoting technological progress to adapt to climate warming at the current level of technology [46]. Japan and Korea exhibit lower ability to use socioeconomic resources to withstand the risk of regional climate variability in rice production. Wheat yields are not significantly affected by climate factors, which is generally consistent with the results of existing studies [46,52]. Furthermore, this research revealed the extent of the influence of major socioeconomic production factors in different wheat cropping regions by dividing the crop regions and showed that the fertilizer application amount per unit area is a common and significant influencing factor of the wheat yields in all cropping regions except the Korea wheat area.

Although the C-D-C model constructed in this paper fits and hindcasts well without considering crop moisture requirements, moisture remains an important ecological factor for the growth of crops and must be emphasized in future studies. In addition, climate change affects not only the yield but also the grain quality. The grain quality is no less important for grain security than the yield and should be emphasized in future research. Whether it is grain yield or quality issues, it always causes negative impacts on society that are valuable to take into account.

IRCC is a new index to assess the impact of climate change on grain yields. Maize yields will be reduced in future climate scenarios in northern China and southwest China. Therefore, it is necessary for northern China and southwest China to choose an alternative crop like rice or wheat that is not significantly influenced or positively influenced by climate change to partially replace maize planting and also to develop advanced technology.

Based on the yield loss time series data, a GPD-based POT model was constructed to calculate the VaR and ES to assess the yield risk in the different crop areas in the current climate state. It has been shown that the grain yield risk is higher in northern China than in southern China [10]. However, this finding varies from crop to crop. For rice and maize, the risk of yield loss is higher in northern China than in southern China, which may be related to the higher risk of drought in the north than in the south [16]. From 2000 to 2015, the affected area and the direct economic loss due to agricultural drought in China showed a distribution pattern of high values in the northeast and low values in the southwest [50]. Although drought-resistant varieties of rice and maize have also been cultivated, their growth still requires sufficient water. For wheat, the risk is higher in southern China than in northern China. This may occur because wheat itself is cold and drought tolerant, and a large number of drought- and cold-tolerant varieties have been generated and cultivated today. Moisture is not a very important limiting factor. Flooding could also cause a wheat yield reduction or even crop failure. From 2000 to 2015, the flood-affected area and direct economic losses in the middle and lower reaches of the Yangtze River were large, with an upward trend [50]. From the perspective of the stability of food supply, the northern provinces of China can expand the planting of wheat and reduce the planting of rice and maize. China’s southern provinces could expand the cultivation of rice and maize and reduce the cultivation of wheat. The north and south can achieve a balance of supply and demand of different kinds of grain in each province through domestic grain trade.

## 5. Conclusions

Compared with most existing studies, this paper provides a more detailed delineation of the crop areas in China for the different crops. The results of this study are more targeted and regionalized. The following conclusions can be obtained:

(1) The effects of climate factors on grain yields vary greatly from region to region and from crop to crop, and the climate environments of regions significantly affected by climate factors tend to be unstable. The rice yields in Japan and Korea are mainly affected by climatic factors, while the rice yields in China are mainly affected by socioeconomic production factors. The wheat yields in China, Japan, and Korea are less significantly influenced by climate factors. Fertilizer application imposes a significant positive effect on the wheat yields in most crop areas. The ability of wheat to withstand the risk of climate change could be improved through rational fertilization. The spring maize area in northern China and the maize area in the Southwest China Mountainous are more affected by climate factors and less affected by socioeconomic factors.
(2) Under future climate scenarios, climate change from 2021 to 2050 exerts a positive impact on the rice crop areas and wheat crop areas but a negative impact on the maize crop areas relative to the climate state from 1991 to 2020. The impact of climate under scenarios SSP1-2.6 or SSP5-8.5 on grain yields is greater than that under scenario SSP2-4.5.
(3) For rice and maize, the risk of yield loss is higher in northern China than in southern China; regarding wheat, the risk of yield loss is higher in southern China than in northern China. This may be related to crop growth habits and the regional climate environment. The risks of yield losses in the Japan rice crop area, Japan wheat crop area, and Korea wheat crop area are relatively high. The risks of yield losses in the Japan maize crop area, Korea rice crop area, and Korea maize crop area are relatively low.

## Figures and Tables

**Table 1:** Division of crop areas.

Crop	Serial Number	Crop Area	Geographical Areas	Growing Period
Rice	1	Double-cropping rice area in South China	Guangdong, Guangxi, Hainan, Hong Kong, Macao, and Taiwan	5–7, 8–10
	2	Single- and double-cropping rice areas in Central China	Jiangsu, Fujian, Shanghai, Zhejiang, Anhui, Jiangxi, Hunan, Hubei, Sichuan, and Chongqing	5–7, 8–10
	3	Single- and double-cropping rice areas on the Southwest Plateau of China	Guizhou, Yunnan, Tibet, and Qinghai	5–7, 8–10
	4	Single-cropping rice area in North China	Beijing, Tianjin, Shandong, Hebei, and Henan	6–8
	5	Early-maturing, single-cropping rice area in Northeast China	Heilongjiang, Jilin, and Liaoning	6–8
	6	Single-cropping rice area in dry area of Northwest China	Xinjiang, Ningxia, Gansu, Inner Mongolia, Shanxi, and Shaanxi	6–8
	7	Japan rice area		7–8
	8	Korea rice area		7–8
Wheat	1	Winter wheat (autumn sowing) area in northern China	Shandong, Henan, Hebei, Shanxi, Beijing, and Tianjin	(-) ^1^ 11–5
	2	Winter wheat (autumn sowing) area in southern China	Fujian, Jiangxi, Guangdong, Hainan, Guangxi, Hunan, Hubei, Guizhou, Yunnan, Sichuan, Chongqing, Jiangsu, Anhui, Hong Kong, Macao, Taiwan, Zhejiang, and Shanghai	(-) 11–5
	3	Spring wheat (spring sowing) area of China	Heilongjiang, Jilin, Liaoning, Inner Mongolia, Ningxia, Shaanxi, and Gansu	5–8
	4	Winter and spring sowing wheat areas of China	Xinjiang, Tibet, and Qinghai	(-) 11–5, 5–8
	5	Japan wheat area		(-) 12–5
	6	Korea wheat area		(-) 11–5
Maize	1	Spring maize area in northern China	Heilongjiang, Jilin, Liaoning, Inner Mongolia, Shanxi, Shaanxi, and Ningxia	5–9
	2	Summer maize area in the Huang-Huai-Hai Plain	Hebei, Tianjin, Beijing, Henan, and Shandong	7–9
	3	Maize area in the Southwest China Mountains	Sichuan, Chongqing, Guizhou, and Yunnan	6–8
	4	Maize area in hilly southern China	Hubei, Anhui, Jiangsu, Shanghai, Zhejiang, Hunan, Jiangxi, Fujian, Guangdong, Guangxi, Hainan, Hong Kong, Macao, and Taiwan	6–7
	5	Irrigated maize area in Northwest China	Xinjiang and Gansu	6–9
	6	Mazie area on the Qinghai–Tibetan Plateau of China	Qinghai and Tibet	6–9
	7	Japan maize area		5–8
	8	Korea maize area		4–8

^1.^ (-) refers to the previous year.

**Table 2:** Threshold selection of each cropping area.

Cropping Area		1 ^1^	2	3	4	5	6	7	8
Maize	Threshold	−31.62	−187.12	−128.88	−38.62	−324.12	−142.19	−27.97	−170.82
	Sorting	16	5	5	19	4	10	5	6
Rice	Threshold	−182.32	−101.21	−275.18	−214.27	−87.64	−313.04	−185.19	−256.75
	Sorting	5	4	3	5	10	5	5	5
Wheat	Threshold	−120.93	−120.78	55.76	−122.59	−237.00	−312.26	-	-
	Sorting	4	6	19	8	7	7	-	-

^1.^ Sorting refers to the number of bits where the threshold is located in the ascending sequence of the data. The crop areas in Table 1 are labelled in order. These labels are used as Table 1 serial numbers.

**Table 3:** C-D-C model test for each crop area.

Crop Area	Residual Sum of Squares (RSS)	Adjusted R^2^	Mean of Relative Error
Double-cropping rice area in South China	0.0710	0.2803	−0.0077
Single- and double-cropping rice areas in Central China	0.0083	0.8972	−0.0012
Single- and double-cropping rice areas on the Southwest Plateau of China	0.0710	0.4274	0.0191
Single-cropping rice area in North China	0.1189	0.6829	0.0341
Early-maturing single-cropping rice area in Northeast China	0.0315	0.7660	−0.0014
Single-cropping rice area in dry area of Northwest China	0.0721	0.8128	0.0114
Japan rice area	0.0661	0.6327	0.0046
Korea rice area	0.0397	0.5443	0.0063
Winter wheat (autumn sowing) area in northern China	0.0270	0.9593	−0.0064
Winter wheat (autumn sowing) area in southern China	0.0853	0.9056	0.0056
Spring wheat (spring sowing) area of China	0.1067	0.7978	0.0090
Winter and spring sowing wheat areas of China	0.0273	0.9445	0.0140
Japan wheat area	0.2909	0.3555	0.0111
Korea wheat area	0.5379	−0.0146	−0.0039
Spring maize area in northern China	0.0876	0.7334	0.0046
Summer maize area in the Huang-Huai-Hai Plain	0.0933	0.6751	0.0069
Maize area in the Southwest China Mountains	0.0578	0.8775	0.0114
Maize area in hilly southern China	0.0704	0.8291	0.0261
Irrigated maize area in Northwest China	0.0994	0.7696	−0.0071
Mazie area on the Qinghai–Tibetan Plateau of China	0.2295	0.4881	0.0150
Japan maize area	0.0027	0.9117	0.0034
Korea maize area	0.0904	0.7355	0.0014

**Table 4:** VIF of each independent variable for each crop area.

Crop Area	*β*_1_	*β*_2_	*β*_3_	*γ*
Double-cropping rice area in South China	1.1945	8.2247	8.6717	1.1898
Single- and double-cropping rice areas in Central China	2.4871	2.2701	2.9919	1.1801
Single- and double-cropping rice areas on the Southwest Plateau of China	3.6948	2.0385	3.0412	1.4064
Single-cropping rice area in North China	1.8717	1.2607	1.6227	1.1130
Early-maturing, single-cropping rice area in Northeast China	2.5946	31.3800	25.0936	1.0502
Single-cropping rice area in dry area of Northwest China	1.9516	1.3012	1.8670	1.3341
Japan rice area	11.4393	9.3500	6.6507	1.0939
Korea rice area	26.2476	17.9801	4.1156	1.0629
Winter wheat (autumn sowing) area in northern China	2.2262	1.4196	2.3437	1.1729
Winter wheat (autumn sowing) area in southern China	2.4213	2.7505	4.2112	1.6201
Spring wheat (spring sowing) area of China	1.3803	4.5462	4.6765	1.1909
Winter and spring sowing wheat areas of China	1.9761	1.0311	1.9356	1.0335
Japan wheat area	6.7006	1.4523	6.6055	1.1486
Korea wheat area	8.1970	4.6529	3.5710	1.0648
Spring maize area in northern China	2.4236	11.7103	9.0062	1.2765
Summer maize area in the Huang-Huai-Hai Plain	5.1883	8.7393	3.0180	1.0175
Maize area in the Southwest China Mountains	4.6703	6.2971	2.6591	1.1282
Maize area in hilly southern China	13.1194	7.6808	4.8837	1.3908
Irrigated maize area in Northwest China	1.6665	9.8974	14.1108	1.8945
Mazie area on the Qinghai–Tibetan Plateau of China	1.4767	2.1175	1.8110	1.3394
Japan maize area	9.0438	3.1719	6.3990	1.0870
Korea maize area	4.1217	2.2174	3.6954	1.1033

**Table 5:** Partial regression coefficients estimated using ridge regression analysis.

Crop Area	Estimation Method	*μ*′	*β*_1_	*β*_2_	*β*_3_	*γ*
Early-maturing, single-cropping rice area in Northeast China	OLS	5.39 *** ^1^	0.36 **	−0.14	0.35 ***	−0.05
	RR (*k* = 0.19)	6.55 ***	0.18	0.05 ***	0.11 ***	0.02
Japan rice area	OLS	7.39 ***	0.07	−0.37	−0.17	0.93 ***
	RR (*k* = 0.08)	7.23 ***	−0.03	−0.24 *	−0.13	0.82 ***
Korea rice area	OLS	7.44 ***	−0.17	0.08	−0.01	0.35 ***
	RR (*k* = 0.15)	8.19 ***	−0.07 **	−0.06	−0.04	0.32 ***
Spring maize area in northern China	OLS	26.64 ***	−0.61	0.15	0.10	−2.86 ***
	RR (*k* = 0.15)	25.62 ***	−0.73 **	0.11 ***	0.12 **	−2.42 ***
Maize area in hilly southern China	OLS	3.99	0.06	0.31 **	0.45 ***	−0.24
	RR (*k* = 0.12)	7.38 ***	−0.18 **	0.21 ***	0.32 ***	−0.17
Irrigated maize area in Northwest China	OLS	8.71 ***	−0.34	0.06	0.36 **	−0.04
	RR (*k* = 0.19)	8.20 ***	−0.14	0.13 ***	0.19 ***	−0.27

^1.^ ***, **, and * indicate that the coefficient is significant at 1%, 5%, and 10%, respectively.

**Table 6:** Partial regression coefficients estimated considering the technological advancements.

Crop Area	*μ*′	*β*_1_	*β*_2_	*β*_3_	*γ*	*b*_1_	*b*_2_
Double-cropping rice area in South China	3.41	0.86 ** ^1^	−0.18	0.13	−0.18	−0.11 ***	−0.04
Single- and double-cropping rice areas in Central China	11.02 ***	−0.19 ***	−0.10	0.16 ***	−0.07	−0.04 *	−0.03
Single- and double-cropping rice areas on the Southwest Plateau of China	16.40 ***	−0.14	−0.42	0.29 **	−1.21	−0.01	−0.11
Single-cropping rice area in North China	9.75 ***	−0.49 ***	0.17	0.41 ***	−0.01	−0.13 *	−0.13 *
Early-maturing, single-cropping rice area in Northeast China	5.47 ***	0.25	0.06	0.23 **	−0.05	−0.03	−0.01
Single-cropping rice area in dry area of Northwest China	5.66	0.13	0.53 ***	0.24 ***	−0.46	0.10 **	0.14 *
Japan rice area	8.14 ***	0.07	−0.48 **	−0.16	0.92 ***	−0.04	−0.02
Korea rice area	7.91 ***	−0.21	0.07	−0.03	0.33 ***	−0.02	−0.03
Winter wheat (autumn sowing) area in northern China	7.26 **	−0.42 ***	0.18	0.53 ***	0.04	−0.01	0.02
Winter wheat (autumn sowing) area in southern China	6.15	−0.81 ***	0.44	0.33 **	0.84	0.00 ^2^	−0.01
Spring wheat (spring sowing) area of China	14.00 ***	0.01	−0.24 *	0.28 *	−1.07	−0.05	−0.04
Winter and spring sowing wheat areas of China	12.50 ***	0.12 *	−0.16 ***	0.44 ***	−1.18	0.03	−0.04
Japan wheat area	12.61 ***	−0.48	0.04	0.60 *	−1.87 *	0.08	0.14
Korea wheat area	12.02 **	−0.72 *	−0.01	0.35	−0.65	−0.13	−0.28
Spring maize area in northern China	25.66 ***	−0.52	0.11	0.09	−2.73 ***	0.01	0.04
Summer maize area in the Huang-Huai-Hai Plain	10.32 ***	−0.32 *	0.17	0.13	−0.24	0.00	−0.02
Maize area in the Southwest China Mountains	7.56 ***	−0.10	0.35 *	0.32 **	−0.59 **	0.05	0.02
Maize area in hilly southern China	3.35	0.23	0.30 *	0.41 **	−0.38	0.06	0.08
Irrigated maize area in Northwest China	9.77 ***	−0.40 *	−0.05	0.37 **	−0.05	0.04	0.10
Mazie area on the Qinghai–Tibetan Plateau of China	19.46 ***	−0.72	0.26 ***	0.02	−1.64	0.00	−0.32 **
Japan maize area	9.02 ***	−0.24 ***	0.06 ***	0.03	0.03	0.00	0.01
Korea maize area	10.56 ***	−0.45 ***	0.02	−0.03	0.07	−0.01	−0.05

^1.^ ***, **, and * indicate that the coefficient is significant at 1%, 5%, and 10%, respectively. ^2.^ “0.00” means the value is smaller than 0.01 and greater than 0.

## Data Availability

The datasets used and analyzed during the current study are available from the corresponding author upon reasonable request or corresponding websites and statistical yearbooks.

## Appendix A

**Table A1:** Fitted curves for the population employed in agriculture.

Provincial-Level Administrative District	Fitted Curve ^1^	R^2^
Beijing	*y* = 112e^−0.024*x*^	0.7410
Tianjin	*y* = −21.11ln(*x*) + 143.01	0.8356
Inner Mongolia	*y* = 3.0086*x* + 451.33	0.9054
Jilin	*y* = 0.0412*x*^3^ − 2.6165*x*^2^ + 49.963*x* + 239.59	0.9399
Heilongjiang	*y* = −0.5616*x*^2^ + 30.485*x* + 274.3	0.7244
Zhejiang	*y* = −28.499*x* + 1532.9	0.9405
Jiangxi	*y* = −0.9503*x*^2^ + 25.921*x* + 914.65	0.8269
Henan	*y* = −0.0496*x*^3^ + 0.5159*x*^2^ + 47.899*x* + 2338.2	0.6863
Guangxi	*y* = 0.0471*x*^3^ − 2.98*x*^2^ + 56.344*x* + 1252.5	0.8693

^1.^ *x* = year − 1981 + 1; *y* is the rural population employed in agriculture (unit: 10,000 people).

## Appendix B

**Table A2:** Model selection.

Model	Institution and Description
ACCESS-CM2	CSIRO (Commonwealth Scientific and Industrial Research Organisation, Aspendale, Victoria 3195, Australia), ARCCSS (Australian Research Council Centre of Excellence for Climate System Science).
ACCESS-ESM1-5	CSIRO (Commonwealth Scientific and Industrial Research Organisation, Aspendale, Victoria 3195, Australia), ARCCSS (Australian Research Council Centre of Excellence for Climate System Science).
BCC-CSM2-MR	Beijing Climate Center, Beijing 100081, China
CanESM5	Canadian Centre for Climate Modelling and Analysis, Environment and Climate Change Canada, Victoria, BC V8P 5C2, Canada
CMCC-CM2-SR5	Fondazione Centro Euro-Mediterraneo sui Cambiamenti Climatici, Lecce 73100, Italy
CMCC-ESM2	Fondazione Centro Euro-Mediterraneo sui Cambiamenti Climatici, Lecce 73100, Italy
IITM-ESM	Fondazione Centro Euro-Mediterraneo sui Cambiamenti Climatici, Lecce 73100, Italy
MIROC6	JAMSTEC (Japan Agency for Marine-Earth Science and Technology, Kanagawa 236-0001, Japan), AORI (Atmosphere and Ocean Research Institute, The University of Tokyo, Chiba 277-8564, Japan), NIES (National Institute for Environmental Studies, Ibaraki 305-8506, Japan), and R-CCS (RIKEN Center for Computational Science, Hyogo 650-0047, Japan)
MPI-ESM1-2-HR	JAMSTEC (Japan Agency for Marine-Earth Science and Technology, Kanagawa 236-0001, Japan), AORI (Atmosphere and Ocean Research Institute, The University of Tokyo, Chiba 277-8564, Japan), NIES (National Institute for Environmental Studies, Ibaraki 305-8506, Japan), and R-CCS (RIKEN Center for Computational Science, Hyogo 650-0047, Japan)
MPI-ESM1-2-LR	JAMSTEC (Japan Agency for Marine-Earth Science and Technology, Kanagawa 236-0001, Japan), AORI (Atmosphere and Ocean Research Institute, The University of Tokyo, Chiba 277-8564, Japan), NIES (National Institute for Environmental Studies, Ibaraki 305-8506, Japan), and R-CCS (RIKEN Center for Computational Science, Hyogo 650-0047, Japan)
MRI-ESM2-0	Meteorological Research Institute, Tsukuba, Ibaraki 305-0052, Japan
NESM3	Nanjing University of Information Science and Technology, Nanjing, 210044, China
NorESM2-LM	NorESM Climate modeling Consortium consisting of CICERO (Center for International Climate and Environmental Research, Oslo 0349), MET-Norway (Norwegian Meteorological Institute, Oslo 0313, Norway), NERSC (Nansen Environmental and Remote Sensing Center, Bergen 5006, Norway), NILU (Norwegian Institute for Air Research, Kjeller 2027, Norway), UiB (University of Bergen, Bergen 5007, Norway), UiO (University of Oslo, Oslo 0313, Norway) and UNI (Uni Research, Bergen 5008, Norway), Norway. Mailing address: NCC, c/o MET-Norway, Henrik Mohns plass 1, Oslo 0313, Norway
NorESM2-MM	NorESM Climate modeling Consortium consisting of CICERO (Center for International Climate and Environmental Research, Oslo 0349, Norway), MET-Norway (Norwegian Meteorological Institute, Oslo 0313), NERSC (Nansen Environmental and Remote Sensing Center, Bergen 5006, Norway), NILU (Norwegian Institute for Air Research, Kjeller 2027, Norway), UiB (University of Bergen, Bergen 5007, Norway), UiO (University of Oslo, Oslo 0313) and UNI (Uni Research, Bergen 5008, Norway), Norway. Mailing address: NCC, c/o MET-Norway, Henrik Mohns plass 1, Oslo 0313, Norway
TaiESM1	Research Center for Environmental Changes, Academia Sinica, Nankang, Taipei 11529, Taiwan, China
